# Realising distributed leadership through measurement for change

**DOI:** 10.3389/fpubh.2023.1155692

**Published:** 2023-08-01

**Authors:** Jonathan Watkins, Nazira Muhamedjonova, Penny A. Holding

**Affiliations:** ^1^HealthProm, London, United Kingdom; ^2^Adjunct Faculty, School of Epidemiology and Public Health, Datta Meghe Institute of Medical Sciences, Wardha, India; ^3^Identitea, Nairobi, Kenya

**Keywords:** responsibility and accountability framework, scaling, paradigm change, decolonisation, measurement for change, monitoring evaluation learning, deinstitutionalisation, vulnerable children

## Abstract

Through a systematic reflection on the journey that transformed traditional state-run baby homes in Tajikistan from closed institutions into community-oriented Family and Child Support Centres (FCSC) we reveal key moments of change. This review describes how community consultation with local participants in a development project shifted responsibility and accountability from international to local ownership and how distributed leadership contributes to the decolonisation of social services. Based on these interviews we ask, ‘How do the innovations of a social development project become a fixed part of normal local social, cultural and political life; and, how do we know when a new normal is self-sustaining at a local level?’ This analysis builds on a network-mapping tool previously described in this journal. Our interviews show that each participant has taken a non-linear journey, building on the networks previously described, under the influence of activities and discussions that emerged throughout the project. We consider how a monitoring, evaluation, and learning process should be responsive over time to these influences, rather than be set at the start of the project. Using the themes that emerge from participants’ journeys, we apply a ‘measurement for change’ (M4C) approach that integrates Monitoring, Evaluation and Learning (MEL) into decision-making. The journey framework applied represents a systematic application of the M4C approach that gives us insight into where local ownership is responsible for the sustainable management of the intervention, and where continued partnership will further strengthen impact and accountability. The exercise has provided evidence of progress towards decolonisation and of the centring of local priorities in MEL and implementation processes.

## Introduction: starting point of the journey

The journey encompasses two dimensions of decolonisation. First, that of cultural and organisational change that reflects local values and moving away from the institutional practises and policies of a former coloniser. Second, the reconstruction of decision making around local accountability and responsibility, moving away from the domination of colonial ideologies determined by Western thought and approaches. The first dimension addresses the legacy of institutional state-centred care from the former Soviet Union. The second involves taking down the ideological and financial scaffolding framed by an International NGO. The challenge of decolonisation we address is the understanding of effective turning points in the transformation from external to local control.

In this paper, we describe the journey of change in Tajikistan from a Soviet model of institutional childcare to a family support model. The journey was rooted in best international professional practises, facilitated by a collaborative programme, Putting Families First (PFF). Using the conceptualisation of a journey we explore how a system involving families, professionals and government becomes locally owned and how information gathering places the local perspective at the centre of the process of change. We document evidence of the transition and describe actions that will continue the journey of scaling effective and sustainable family based support.

In Tajikistan, recently independent from Soviet governance, it remains common for children to grow up in extended families, where fathers take most decisions and fathers’ mothers (mothers-in-law) retain a strong influence over daughters-in-law and grandchildren. Family centred support is therefore a more traditional model than that imposed in the Soviet era, when children in need were cared for in institutions, known as Baby Homes. As a low-middle income economy many adults, particularly men, emigrate for Russia to seek employment, and absent parents can leave families struggling to meet the needs of their children. Support systems have provided limited resources directed at child protection and family support, either from the state or other entities. The process of transition from an institutional to a family support model here described is a continuing process.

Under a centralised institutional system of child support, accountability and responsibility for care is taken from the family and given to a very narrow group of actors. Centralised decisions about children are remote from the family unit and filtered through multiple administrative levels resulting in a slow processes of change led by fixed requirements of the service, not the needs of the child. The initial theory of change for PFF, developed as a collaboration between INGOs, funding partners and local service providers, was directed at broadening the accountability and responsibility framework and for decisions to be more child and family-centred. It recognised that changing from centralised support to family centred care requires change throughout the system, which Meissner refers to as ‘Alignment’ (1). The goal was, is, to return the central role of childcare to the family supported by services that create shared accountability and responsibility. Gaining national and local government support for this transition, and parental trust in new support structures was crucial and a primary focus of activity in the process of transformation.

We review the changing structure of the decision-making process using the approach of Measurement for Change, M4C (2, 3). M4C highlights, through five overlapping and interconnected aspirations, key components of information systems that build effective decision-making, by making data accessible to, and useful for, all participants in the network of practise and support. We also reflect on the contribution the PFF journey makes to the wider understanding of decolonisation and the central role that data play in establishing local ownership and local relevance. Progression from one stepping-stone to the next on the journey has been marked by moments of realisation, the ‘А бача! (*A bacha*!)’, a Tajik expression of surprise and delight when an idea or concept emerges and makes sense in context. In this paper, we discuss the pathways and the *A bacha*, through which the rights and needs of children are met locally. Like the Silk Road, these pathways are networked, multi-faceted and change over time (4).

## Monitoring and evaluation using M4C

### Methodological framework

Integrated into the journey towards sustainable and effective delivery is the transformation of decision making from a top down hierarchical process to one that reflects distributed responsibilities through shared accountability. The foundations of this approach are closely aligned to Parker-Follet’s conception of integration and collaborative, shared leadership (5). These conceptualisations have continuously emerged and re-emerged in organisational, management and leadership theory since first being published in the 1920s (6–8). In the process of decolonisation M4C embeds the principles of Diversity, Equity and Inclusion^1^ into the collection and use of information.

Information plays the key role of drawing the network of participants into the conversation on design and implementation. Diversity means the drawing on multiple sources; Inclusion, the recognition of multiple perspectives, and Equity, the attribution of comparable weight to different values. The opportunity to contribute and participate in information based decision making stimulates the transformation. At the heart of this transformation in PFF lay building the capacity of the implementing partners to monitor, evaluate, and learn. Aligned to the M4C aspirations, an MEL system that applies the principles of shared listening and learning using participatory methods was key to achieving this objective. Experience was created in partners of information collection and utilisation methods that are consistent, rigorous, systemic, feasible, and supply useful information in context (4).

### Objectives

The focus of this paper is the process of reflective practise applied post-external funding to uncover the key components of the programme that held value over time. We were interested in clarifying what is happening now, and how this relates to the turning points. This stage of reflection builds on the experience of information gathering from earlier stages of the journey, an example of which involved participants in mapping the ecosystem of support for childcare. The details of this exercise are shared in an earlier publication (4). The exercise identified systematic consultation as a valued process as well as three key areas of action: (1) Regular consultation. Discussion sessions to share experiences and learning between the families and support services, and across families, to build trust and networks of support; (2) Targeting fathers. Bringing fathers more intentionally and directly into the Family Centre services to strengthen the childcare system; (3) Directed support for mothers with restricted support networks. The triangulation of data from mapping, systematic Observations of Mother Child Interaction (9), and case management notes, guided support staff to stimulate positive bonds between the more isolated mothers and their children.

### Process and tools

We here describe uncovering the experience of the readiness of component activities to scale, the capacity of the information system to improve the delivery of quality nurturing care (10), and the shift towards local responsibility and accountability. We report on an interview-based structured reflection process applied in a series of individual in-depth interviews.

The interview process itself was developed in stages. The parameters of interest were clarified by the authors (JW, NM and PH), who also listed potential questions relevant to a conversation around opportunities, responsibility and accountability. These were refined through an initial interview to focus on stimulating individual reflections without being overly prescriptive around the themes and topics that might emerge. The questions/prompts asked were:

From your point of view, how is the project ready for scaling up?
How can you determine that the project is ready for scaling?
What lessons have you learned from the funded project?
How important is partnership in the development and effectiveness of the project?
What changes have occurred as a result of the funded project?
What tools would you recommend using in a new project?
In which areas of life do you think Children With Disability are not fully included or given opportunities?
What is the area where you ultimately hope to see change?

Permission to carry out the cycle of evaluation came from the Ministry of HSPP (in full) as part of their ethical overview. Interviewees provided individual consent to participate, having been informed of the purpose and process of the reflection. Personal identifiers were kept separate from interview scripts.

### Study sample and sampling

Interviews were carried out by author NM, who also took responsibility for identifying the respondents and recording the data. These were carried out with service providers, as, in light of discontinuity in funding, and with key decision-making moments falling outside the funding cycle, we were unable to include reflections from across the whole network of support in one step. The perspectives of Hayot dar Oila and Sarchashma, the two NGOs who manage the development and delivery of the family based services, were each represented by four respondents. These eight interviews included three managers (NR, ZN2 and UE) and five service providers (ZN1, FB, DM, SS and ZP). Two additional respondents represented the perspective of the Baby Homes, now re-fashioned as FCSCs. In Dushanbe the respondent was a social worker (FB), and in Khujand the Director (SS), both had been with the project since its inception in 2006. Respondents were selected to represent the breadth of experience of those who had been on the journey during the development and implementation of the PFF programme.

### Analysis

The information was collected, reviewed and fed-back in the original in Russian and Tajik. English translation of the material was only completed to support discussion amongst the authors in the development of a summary framework, and to share key examples with the readers of this paper. The subsequent interviews were harvested for the *A bacha*, the reflections that bring learning of the impact on participants and the system of support. NM carried out the initial review of responses. The initial grouping of responses was discussed with JW. The thematic organisation of examples was then reviewed by PH, and any suggested changes discussed with the other authors prior to production of a final summary list.

Once individual themes were extracted a journey metaphor provided a framework to display and discuss the characteristics of change that emerged through the conversations with respondents and between the authors. The methodology itself developed out of team reflections on individual children’s life journeys, in which key events and experiences can be linked to changes in the direction of a child’s development. Here, we have adapted this idea to track observable changes in the system of support. The journey of PFF is described through relating shifts in accountability and responsibility to emerging influences, related events, and their consequences, pursuant to each project milestone. The intention is to reveal how decision-making, ownership and data sharing has changed over the course of the life span of the intervention.

## Results: the learning

### Reflections and the *A bacha*

The reflections shared in Supplementary Figure 1 are drawn from 10 interviews carried out in 2022. All respondents were female.

The reflections illustrated:

Changes in the system of support: in resources available, access to and utilisation of services.
Changes in responsibilities/relationships: growth in self-awareness, confidence, trust, job satisfaction and professionalism.
Changes in accountability: in the use of information to monitor, evaluate and learn.
Scaling the system: current gaps and recommendations to build future steps.

Interviewees told us that, through this project, they came to understand that institutional care is harmful for children and that a model of care that emphasises both social and medical support is effective. Their motivation for change grew from a combination of knowledge and understanding of early child development, the experience of changes in practise and the building of trust within the network of support. All interviewees commented that they now worked more closely with other professionals, indicating a move towards group responsibility for child and family care. Equally, they observed a greater inclusion of parents in therapeutic care and a transfer of responsibility to parents for meeting the daily needs of the children.

The evidence base and the collecting and using of data were central to the process of building trust. The tools they used for measuring change and evaluating needs gave them the information they needed to target and improve their services. We also heard that information from monitoring and evaluation was used to explain to senior decision-makers the value of the project innovations. Initially strongly influenced by the requirements of external funders to report on progress and impact, the implementation team developed a growing awareness of the value of information in guiding forward planning to meet individual needs as well as to influence wider policy and practise.

### The journey, summary narrative

The journey is set out in Table 1 where time-related markers are associated with influences, events and a description of the consequences of each event alongside the progressive handover of responsibility and accountability.

**Table 1:** The journey.

Years from the start	2006	2008	2009	2011	2012	2013	2016	2019	2020	2022
Milestones reached	Foundations for change adopted	Partnerships and networks forged	Culture change shared goal	Increased advocacy for change	Gap in international funding	Engagement with innovation consolidated	New funds secured	Focus on evidence building	Internal funding	System sustained
Emerging influences	Inter-agency trust	Social and integrated care	Social inclusion	Child development knowledge	Local autonomy	Multi-sectoral trust	Measurement for change	Policy and legislation enacted	FCSC model	Self-belief
Related events	Approval by City Health Department	Implementation processed initiated	Local provision for children with disabilities	Service innovations prioritised	Partnership models adopted	Family centres prioritised in all baby homes	Family support model dominates	FCSCs replace baby homes in law	Local laws passed	NGOs support best practise
Consequences	Partnership of Dushanbe City Health Department and INGO	Engagement in professional development	Inclusion of children with disabilities	Local family centres opened	Growth in demand for community support	Institutionalisation decreases	M&E more intentional	Social model becomes mainstream	Community care local government norm	Focus on long term sustainability
Responsibility	Government, INGO	Government, INGO, NGOs	Local government and parents consulted	Baby homes broaden responsibilities	Service delivery staff	Multi-agency partnerships	Shared leadership includes parents	Parents’ role expands	Local government directs family support	Distributed leadership
Accountability	INGO led data informed fund raising	INGO NGOs reporting to funders and government	Exploration of impact on children	Service providers report to parents	Internal reporting systems in NGOs	Multi-agency evaluation of service provision	NGOs use M4C approach to MEL	System for data sharing in place	Local government legally accountable	Functions at multiple levels

The journey towards community supported child care in Tajikistan began in 2006 when the Dushanbe City Health Department asked an INGO, HealthProm, to identify alternatives to supporting children in need in baby homes. The PFF collaborative programme facilitated the transformation of child support from a Soviet-legacy institutional model into a family care model that reflects traditional Tajik values. As can be seen from Table 1, the Journey has covered an extended period, not consistently supported by external funding. It started with the essential foundations of partnership building across government and non-government agencies, introduction of innovative social work and therapeutic professional practises.

Until 2007, disabled people received mainly a medical model of care. Such services as occupational therapy and physical therapy began to be introduced for the first time in the post-Soviet space through this project. On the site of the first baby home, the first early intervention centre was opened with the support of HealthProm. In 2011, with funding from the European Union and United Kingdom Aid, technical assistance was provided to expand skills and develop policy and practise in social protection. The project began to introduce tools to assess changes in the development of children. In 2017, this process became more structured under the funding of Grand Challenges Canada, with the introduction an MEL system guided by M4C.

Self-awareness, confidence, trust, job satisfaction and professionalism all built slowly over time. Thirteen physical therapists and 15 occupational therapists were trained under PFF to move beyond the provision of massage and electrical therapy. At the beginning, it was difficult for innovations to be accepted. There was little trust in the relations between therapists who provided new improved services and the doctors in medical services. However, the positive results arising from PFF have changed the attitude of doctors. Doctors began to refer their patients, and the skills of these specialists have become recognised by the community. In the first years of the early intervention centres, parents complained that they had brought their child not for play but for treatment. Later the realisation grew that play is a key element in the learning and development of children. In subsequent years, when the community saw positive results from the intervention, many more children came. Many families were referred by polyclinics and many came by themselves, as they heard from other parents about the positive results of the interventions. During the course of the journey of change a community model of support has become embedded in national policy and local law. Community based services are now accessible to those families that reside within easy travelling distance of the four FCSCs, operating from what used to be the Baby Homes. Whilst the funded programme that actively contributed to the establishment of the FCSCs has ended, the centres continue to operate under Local Government Authority management and families remain engaged within the new system of support.

At the start of project work in 2006 key responsibility for design and resources were remotely located with the INGO. The government of Tajikistan, in the process of reclaiming a national identity after some 80 years of Soviet influence, was a key partner, motivated by a general wish to comply with the UN Convention on the Rights of the Child and achieve the Sustainable Development Goals. Over the lifetime of the project work, and following a series of *A bacha* moments that arose out of evidence, increased acceptance and engagement has followed. Responsibility for project management and delivery became progressively more local. The Local government Authorities where the former Baby Homes were located took ownership of the new identity of the FCSCs by passing by-laws that ended the institutionalisation of young children and legitimised a community care model of family and child support. Responsibility for implementing and sustaining best professional practises has passed progressively from the INGO to local NGOs. Local NGOs assumed responsibility at an early stage of the project for advocating for change, sharing their skills and knowledge with other professionals, and ultimately assumed a MEL role to ensure sustained quality in the Local Authority run FCSCs. They remain with the responsibility of developing local skills and knowledge for the care of vulnerable children in their families.

Accountability, the evidence that supports effective delivery also shifted from a focus on information collected to address the values of international actors and towards local audiences. The locus of control has moved closer to families, the ultimate beneficiaries. The major shift in accountability came during 2019 when the non-state sector, the INGO and local NGOs, passed control of the FCSC to Local Government Authorities. New by-laws meant that Local Governments now managed the family support services and assumed accountability to the local population for the quality of care provided through the democratic systems of local governance.

In 2020, the vision of distributed leadership was achieved to the extent that local government assumed authority for service management, local NGOs for quality assurance, national government for service specification and parents for the care of their children.

## Discussion/reflection

The transformation of Tajikistan’s closed baby homes into family centres has provided innovative multidisciplinary community-based care, depending on trust between parents and services. We became aware of, and built on, the close connection between trust and evidence, which is echoed in the Russian proverb, ‘доверяй, но проверяй’ ‘trust but verify’. From the start, the PFF project has had a paradoxical relationship to decolonisation. Whilst raising questions about the value of state parenting and reviving a traditional focus on the family, it has also used external (Western) approaches and resources to scaffold structural change. The decolonisation of institutional care has had two phases. That of replacing closed institutions with open community-oriented family support has been largely completed. Removing the scaffolding of the PFF internationally funded action continues to shift the responsibility and accountability for care towards those more directly connected to vulnerable children, the families, local authorities and national policy makers. The theme of trust permeates the overall journey from initial building of partnerships and forging new networks, to creating a culture of change where participants are willing to entertain new concepts and practises.

The journey framework illustrates how trust developed over the long term, and the narratives illustrate the central role trust plays in building the relationships necessary for change to happen. Trust created conditions of openness to new ideas, loosened the ties with established patterns of care and enabled the development of responses to changing circumstances. In this journey, the role of an INGO is to work consistently to build trust and self-confidence between state and non-state partners, allowing for the possibility of innovation to be introduced. The INGO had also to trust the relationships built to shift the framework of responsibility and accountability to more local control.

In spite of introducing uncertainty and discontinuity, the cyclical transition between periods of international funding and unfunded phases, has provided space and released local actors to cement, integrate and localise their ideas and practises. We observed a pattern where innovations happen occasionally and periodically, rather than smoothly over time. Changes to the status quo did not take place in direct response to new experiences and opportunities. Rather, a critical mass of evidence needed to build up to create the *A bacha* moments that punctuate the journey, marking the ratcheting-up of project innovations towards system change and sustainability. Examples of alignment of the system into new best practises include the realisation that social family support, rather than clinical vitamin injections lead to improvements in child development, as well as the passing of a by-law ending young child institutionalisation by the first local government authority. Professional attitudes changed in response to pressure from parents, requiring also the influence of widespread training, coaching and the lived experience. Policy change occurred when local NGO leaders gathered and then disseminated evidence of the impact of the innovation. We observed that a new status quo or paradigm emerged when pressure for change built, and people let go of familiar ideas to embrace personal change. These examples reflect both Thomas Khun’s model of paradigm change (11) and Karl Popper’s notion that change happens when people change (12). An extreme example of the Popper notion was when family support services only developed in one Baby Home after there was a change of Director. As suggested by M4C, data played a central role in driving conceptual changes in the paradigm. Evidence provided micro-steers to those who gathered the data, and for those with whom it was shared. The journey is open-ended because the evidence is still building, and changes achieved so far will, in time, be overturned by new knowledge, and by new generations of practitioners adapting to specific events and changing local circumstances (12).

The M4C approach contributed to the decolonisation process by asking the question, ‘To whom does the data belong and for whose benefit is the data collected?’ Data are valuable more than for its quantification or qualification of actions and events. It empowers participants who draw on it for everyday use. Whilst the project has used data to demonstrate that externally set project targets have been met, and to contribute to and demonstrate wider objectives, such as the Sustainable Development Goals, we also recognise that one-time evaluations have consistently failed to generate sufficient information to support the transformation into sustainable systems (13). The data we report on in this paper demonstrates that for data to be useful, and used, it must be based upon the needs and circumstances of all participants, and feed back into their lives. The dynamic use of data interacted with the project as a whole to direct frequent ‘micro-steers’, and feedback loops. As an example, conversations around networks (4) revealed the absence of fathers from day to day care of children, triggered PFF to strengthen the parenting programme for fathers.

In the process of decolonisation, evaluating impact and creating sustainability and feasibility at scale requires the MEL system to reflect the priorities of participants central to the intervention, perhaps, more so than the needs and values of those external to the process of change. To create change requires MEL also to be innovative (2). These have also been our experiences. The implementation team, largely applied practitioners, began with limited experience of managing and utilising integrated monitoring and evaluation systems. Professionals and decision-makers were wary of the potential for measurement to be used to judge the quality of their work, and feared losing face in the sight of more senior managers. We also experienced resistance from the government to developing a monitoring and evaluation system, whose primary role might be to publish impact internationally. These attitudes reflect a not uncommon blame culture, rather than a learning approach. As the project progressed the value of the information gathered grew alongside an increasing appreciation of rigorous and systematic data systems. The implementation team built awareness of the interconnection between developing the skills to track and measure change and the value of the information collected to improve communication and decision-making. A transition from data being used to deliver instructions to data informing collaborative learning was achieved. We have also observed that an extended time frame was required to build the capacity to generate detailed longitudinal data on programme impact.

Recommendations made by the local practitioners for future steps addressed further building of skills and the sharing of those skills with other professionals. It also stressed the need for continued close collaboration with government agencies. Government involvement in the planning and design of new programmes was seen as key to establishing sustainability and scale. They also stressed the multi-departmental nature of the network of support required, inclusive of the ministries of health, welfare, and education. In this conceptualisation, there remains a role for all partners to build and share skills. Commitment to distributed leadership and rigorous implementation, to trust and verify, will continue to strengthen the quality and impact of the innovation as it scales.

## Conclusion and main learning points

The conceptualisation of the journey of transformation identified the cyclical route of awareness, experience and learning through which the turning points emerged. Awareness of the implementing partners of the valuable contribution to effective decision making of regular, systematic Monitoring and Evaluation transformed the engagement with quality practise. Experience in applying different data collection tools and methods transformed the capacity of the team to drive their own professional capacity building. A critical mass of information collated stimulated the learning that led to implementation redesign, and to policy formulation. In each of these turning points a shared process expanded awareness, experience and learning across the network, driving still further forward the journey towards sustainability.

## Data availability statement

The raw data supporting the conclusions of this article will be made available by the authors, without undue reservation.

## Ethics statement

The studies involving human participants were reviewed and approved by Ministry of Health and Social Protection of the Population, Tajikistan. The patients/participants provided their written informed consent to participate in this study.

## Author contributions

JW, NM, and PH contributed equally to the development of the process presented in this paper. All authors contributed to the article and approved the submitted version.

## Funding

This paper reports research done in the Putting Families First programme in Tajikistan. The research contributing to this paper was initiated in full and funded in part by Grand Challenges Canada (Grant Number 1707-08354). The project as a whole is funded by EU Aid (Grant Number ACA/2016/375-595) and United Kingdom Aid (Grant Number 54HT-Q3FN-QY) as well as Grand Challenges Canada. The role of each funder is to finance and monitor the project objectives.

## Conflict of interest

The authors declare that the research was conducted in the absence of any commercial or financial relationships that could be construed as a potential conflict of interest.

The handling editor ZS is currently organising a research topic with the author PH.

## Publisher’s note

All claims expressed in this article are solely those of the authors and do not necessarily represent those of their affiliated organizations, or those of the publisher, the editors and the reviewers. Any product that may be evaluated in this article, or claim that may be made by its manufacturer, is not guaranteed or endorsed by the publisher.

## Footnotes

^1^https://thedecisionlab.com/referenceguide/organizational-behavior/diversity-equity-and-inclusion-dei downloaded 25.5.23

## Abbreviations

DIIS: Dynamic, inclusive, informative, interactive, people-centred
FCSC: Family and child support centre
MEL: Monitoring, evaluation, learning
M4C: Measurement for change
OMCI: Observation of mother child interactions
PFF: Putting families first
