# Supplementary material for: Realising distributed leadership through measurement for change
Source: Front Public Health. 2023 Aug 1;11:1155692. doi: 10.3389/fpubh.2023.1155692 (PMC10427148; doi:10.3389/fpubh.2023.1155692)
Supplement: Supplementary file 1 [file Data_Sheet_1.docx]

**Supplementary Material**

**Supplemental Figure 1. Reflections and *A bacha***

**Changes in the system of support (examples of changes in; the resources are available, access to these services, awareness the rationale and of users that these services are available).**

1. *At the beginning of the project, I personally learned how important it is for a child to have a loved one from birth to three years old, how children's homes affect their development and their future life.* ***ZN1, NGO physical therapist****.*
2. *The essence of a family-centered approach in working with children is important* ***NR, NGO,manager****.*
3. *The project will be ready for completion when the child protection authorities. Hukumats. the ministries of health and education realise that closed child care facilities are detrimental to the development of children.* ***ZN1, NGO physical therapist.***
4. *Orphanages have been transformed into Family Support Centres, community-based early intervention services are provided, day care staff are trained to provide services, parents are trained in RC skills, the number of children living in day care centres has decreased by providing day services, returning children to biological families and preventing children from entering daycare centres.* ***ZN2, FCSC, Manager.***
5. *The newly created services within the transformed orphanages have clearly increased the coverage of vulnerable children, including children with disabilities and developmental delays, with early childhood development, early intervention and other social services. The services of the CPSR are used not only by residents of the cities where the centres are based, but also by residents of other nearby areas. Another indicator of the effectiveness of the provision of new services is the fact that fewer and fewer children are left without parental care. The referral system for vulnerable families began to work better. Families learn about existing services at health centres, local hukumats, community organisations, and other parents. Information about existing services is also provided to families in the form of bulletins, which are distributed to them by family doctors during a scheduled family visit. The poorest families with young children have access to free services. Services are accessed by children with disabilities living in remote rural areas who cannot attend the centre but can receive services at home* ***UE, NGO Director and in-country project lead.***
6. *There are four orphanages, two in the Sughd region and two in Dushanbe. All orphanages operate according to the new system: Functioning mother's room with a child; Department of early intervention; Group "respite care”; Day stay group; ”Short stay" group; Temporary stay department; Five day stay group; Long stay group* ***FB, FCSC, Social worker.***
7. *Big changes. BH moved from a closed institution to an open one. They began to work more with families and support them. They used their services, became mentors to other specialists. The number of children with disabilities, in particular with cerebral palsy, increased , after our services, they went to ordinary schools. Dilafruz,PT,Marvorid*
8. *Orphanage transformation happened? Because of PFF and previous projects*  ***NR, NGO,manager.***
9. *There became confidence in its own importance. Inclusion and integration went forward. We were able to do it!* ***DM, FCSC, physical therapist.***
10. *Inclusion project with befrienders. Before this project, children with disabilities hid and were not participants in this life.* ***SS, Deputy Director former Baby Home Khujand.***
11. *Global changes occurred during the transformation of orphanages. Caregivers, health professionals, teachers and parents have learned a lot about early development, earlier intervention, learning by means of the game, rehabilitation by an active method (PT and OT, development of the strengths of the child)* ***FB, FCSC, Social worker.***
12. *All changes and innovations are not immediately accepted and implemented, it is a long process that takes time to understand, accept and implement.* ***ZN2, FCSC, Manager.***

**Changes in relationships that show changes in responsibility in the people in the system (examples of growth in self-awareness, confidence, trust, job satisfaction and professionalism).**

1. *PFF has helped many children return to their biological families. We support families and children stay in the family. No child care institutional can replace the family.****SS, Deputy Director former Baby Home Khujand.***
2. *Representatives of public services working with vulnerable families and children, repeatedly at meetings, noted that they would like to create the same services in their areas. Representatives of the MOHSP noted several times at official meetings that the transformation of orphanages into family and child support centres and the creation of new services to support vulnerable families and children is one of the great significant achievements in the system of early childhood development and early intervention. -* ***UE, NGO Director and in-country project lead.***
3. *At first, relations between those who provided the new social services of Physical Therapy and Occupational Therapy were not trusted by physicians. But I am recognised by doctors now.* ***DM, FCSC, physical therapist.***
4. *Partnership helps a lot. How to work without a partnership? Learning from the experience of others and implementing the experience of partners This helps in the development of our work* ***SS, Deputy Director former Baby Home Khujand.***
5. *Partnership and interdisciplinary approach and interdepartmental is very important for effective development.We are like a chain dependent on each other. For the development of the child, it is important to know the rights of the child and to educate and raise a healthy child.* ***FB, FCSC, Social worker.***
6. *Partnership is important in improving professionalism and knowledge* ***DM, FCSC, physical therapist.***
7. *I learned case management and how to work with children I always doubted at the end of the project whether the staff of the orphanage would be able to continue our work Worried about where the parents would go if we stop working Now I feel that they have learned a lot and can continue our protect by themselves* ***ZP, FCSC, Psychologist.***
8. *I began to raise children taking into account the knowledge gained****NR, NGO, manager.***
9. *Use knowledge in life. Experience of how to build relationships in the family. All this knowledge was new and I use it to this day in my life* ***SS, Deputy Director former Baby Home Khujand.***
10. *I was very happy with the joy the children went home when their relatives came for them.* ***ZN1, NGO, physical therapist.***
11. *I understood the importance of Early child Development and early intervention, the timeliness of the provision of services, acquired management skills, gained rich experience and knowledge from international experts.* ***ZN1, NGO, physical therapist.***
12. *Training other organisations contributed to the growth and improvement of their knowledge* ***DM, FCSC, physical therapist.***
13. *By virtue of my profession (paediatrician), I used to pay attention only to the disease and treat the patient with medicines. After participating in the project, I realised how important early detection and early intervention is for children with various developmental disabilities.* ***ZN2, FCSC, Manager.***
14. *This project has a very big impact on me. I am a psychologist before school age and the project gave me the opportunity to develop in this area and thanks to this project I love my job.* ***FB, FCSC, Social worker.***
15. *The project makes possible to socialise the child and adapt to the environment. Parents are trained and for one thing there is an opportunity to find time for their needs and work.* ***FB, FCSC, Social worker.***
16. *Many children began to walk, improvement in all areas. Seminars on feeding, on PT, ET. Prior to the project, there was no knowledge on these topics* ***SS, Deputy Director former Baby Home Khujand.***
17. *I got a lot of knowledge regarding how to help to children with autism. There are a lot of children with autism now in our country.* ***DM, FCSC, physical therapist.***
18. *I think the project will be ready for distribution until our higher officials change their perception of the importance of having a child in a family. At the end of the project, many children were returned to their families home, as the employees worked a lot with their parents, conducted trainings, the Mature Parenthood program, where they began to understand how orphanages affect the health of their children and their further development and life. I believe , which is very important to continue partnerships in the development and effectiveness of the project, since children's institutions often change employees who need to be trained and control the work in the centres.****ZN1, NGO physical therapist.***
19. *Without establishing partnerships with government agencies.* ***ZN2, FCSC, Manager.***
20. *The potential of specialists providing services is increased and improved every year. Specialists learned how to work in a team, apply an integrated, family-centred approach to working with vulnerable children, learned how to properly maintain new documentation, expanded cooperation with other service providers, and attracted community resources. Social workers try to prevent the separation of children from their parents, and for this they try in every possible way to help the family at a time of crisis (they provide psychological support to parents, attract other resources, for example, find temporary housing for the family, provide food for the child, etc.).* ***UE, NGO Director and in-country project lead.***

**Changes in accountability, in how people have used information to monitor, evaluate and learn (examples of the feedback loop, the use of tools in the process of change.**

1. *Early intervention Timely assistance in the development of the child after monitoring by Zelinsky. Not costly tools and we find the problem in a timely manner and help in the development of the child.* ***SS, Deputy Director former Baby Home Khujand.***
2. *Currently, the centres mainly use case management technology tools in the process of assessing the needs of the child and family, planning care and tracking progress in the development of the child and solving difficult family situations. If necessary, specialists also use additional forms of assessment to obtain more complete information about the child's condition. The centres work closely with parents to increase the capacity of parents to care for and develop their children. Raising the potential of parents occurs through individual and group consultations, theoretical and practical training of parents. The opinion of parents is taken into account in the process of developing a child development plan.* ***UE, NGO Director and in-country project lead.***
3. *Case management. Monitoring of child development These tools helped in goal setting, effectiveness of rehabilitation as well as family support.* ***DM, FCSC, physical therapist.***
4. *Observation of mother-child interaction helped a lot in assessing attachment .This tool helped us to improve our mother-child relationship skills. Helped a lot in assessing relationships.* ***DM, FCSC, physical therapist.***
5. *While working in the field of physical therapy (PT), I noticed that the population receives services and is informed about РТ and see the results There is a comparison with other services. The outlook on life for children and the family has changed.  Relations with the family have improved. Used professional knowledge in raising our own children ,as I told before it helped with the relationship with the families’. Personal professional growth appeared. Gained experience, became a mentor recognizable, Doctors began to refer patients.* ***DM, FCSC, physical therapist.***
6. *Other organisations see an improvement in rehabilitation (using data form) Zelensky. Everyone saw the benefit and the result; many inspectors said why it is not distributed in other organisations. new method and move forward* ***SS, Deputy Director former Baby Home Khujand.***

**Scaling the system - current gaps and recommendations to build in future steps**

1. *It is very important to work hand in hand with employees of public institutions N*
2. *Partnership in the development and effectiveness of the project is very important without the support of the Ministry of Health, it would be difficult.****NR, NGO, manager.***
3. *Understanding the changing of government officials is very important.* ***NR, NGO, manager.***
4. *For the sustainability of the project and the continuation of activities, it is necessary to support government agencies, public funding.* ***ZN2, FCSC, Manager.***
5. *To what extent are government agencies ready to take full responsibility for themselves (financing, training of specialists, training of parents / guardians, guaranteeing the continuity and quality of services provided, customer satisfaction with the result).* ***ZN2, FCSC, Manager.***
6. *Understanding readiness and ability of state structures to continue activities.* ***ZN2,FCSC, Manager.***
7. *Inclusion is important for child with disabilities but not every institution is aware of this. We need to develop this system. Let there be more projects like this. Children are our future.* ***FB, FCSC, Social worker.***
8. *The project is already scaling, but I would ask a question of the educators of children's institutions and schools, are they ready to work with children with disabilities or not?* ***FB, FCSC, Social worker.***
9. *We need to put lessons in colleges and universities so that there are no gaps. The project is already spreading and our partners from Great Britain, Healthprom, Soros foundation, UNICEF, MINISTRY OF HEALTH, CRU and Ministry of Education, etc. have helped us in this.****FB, FCSC, Social worker.***
10. *We need to develop Physical Therapy and Occupation Therapy, because these services brought a lot of change in the lives of children with disabilities. It helped to be more independent.* ***SS, Deputy Director former Baby Home Khujand.***
11. *I believe that the transformation was successful, did not require additional funding, and it is necessary to disseminate this experience to other regions of the country. These regions can be selected by conducting a needs assessment for local population services and by examining the existing capacity and readiness of local governments for the proposed changes.* ***UE, NGO Director and in-country project lead.***
12. *Building multidisciplinary teamwork of the centre from which a lot of help comes* ***DM, FCSC physical therapist.***
13. *To scale up new services, as Physical therapy and Occupation therapy and want other centres to work in the same direction, is important* ***DM, FCSC, physical therapist.***
14. *Many children with speech impairments. We need training in this area, learn a  new methods.* ***ZP, FCSC, Psychologist.***
15. *Preparation for school is going well on our part, but the teachers and teachers at the school are not prepared They do not know the intricacies (nicety) of working with children* ***DM, FCSC, physical therapist.***
16. *We need to support vulnerable families with disabilities more. We can see financially well off families making progress faster.* ***DM, FCSC, physical therapist.***
